# Meta-analysis of the efficacy and safety of Ginkgo biloba extract for the treatment of dementia

**DOI:** 10.1186/s40780-015-0014-7

**Published:** 2015-04-10

**Authors:** Masayuki Hashiguchi, Yuriko Ohta, Mikiko Shimizu, Junya Maruyama, Mayumi Mochizuki

**Affiliations:** Division for Evaluation and Analysis of Drug Information, Faculty of Pharmacy, Keio University, 1-5-30 Shibakoen, Minato-ku, Tokyo 105-8512 Japan; Department of Hygienic Chemistry, Faculty of Pharmacy, Keio University, 1-5-30 Shibakoen, Minato-ku, Tokyo 105-8512 Japan

**Keywords:** Meta-analysis, Ginkgo biloba extract, Dementia, Efficacy, Safety

## Abstract

**Background:**

The benefit of Ginkgo biloba for the treatment of dementia remains controversial. The aim of this study was to evaluate the efficacy and safety of Ginkgo biloba in patients with dementia in whom administration effects were reported using meta-analysis.

**Methods:**

We searched MEDLINE, Embase, the Cochrane databases, and Ichushi for controlled trials of Ginkgo biloba for the treatment dementia. Clinical characteristics and outcomes were extracted. Meta-analysis results were expressed as standard mean differences (SMDs) in scores of the Syndrome Kurztest (SKT), Alzheimer’s Disease Assessment Scale Cognitive Subscale (ADAS-Cog) for cognition efficacy, or odds ratios (ORs) for dropouts and adverse drug reactions.

**Results:**

Thirteen studies using the extract EGb761 met our inclusion criteria, which were duration of 12 to 52 weeks and daily dose of more than 120 mg, and included a total of 2381 patients. Meta-analysis was performed by using 9 of 13 studies, 7 of which used the SKT and 2 ADAS-Cog (dose 120 mg, 26 weeks) scores as efficacy parameters. In meta-analysis of all patients, SMDs (95% confidence interval [CI]) in the change in SKT scores (7 studies) were in favor of Ginkgo biloba over placebo (SMD = –0.90 [–1.46, –0.34]), but 2 studies that used ADAS-Cog did not show a statistically significant difference from placebo for ADAS-Cog (–0.06 [–0.41, 0.30]). For Alzheimer’s disease (AD) and vascular dementia (VaD) subgroups, SMDs [95% CI] in SKT in the combined AD and VaD subgroup (–1.07 [–1.66, –0.47]) and AD subgroup (–1.36 [–2.27, –0.46]) were in favor of Ginkgo biloba over placebo. In terms of daily dose of Ginkgo biloba in the combined AD and VaD subgroup, SMD in SKT score in 240-mg daily dose groups was significantly greater than with placebo (–0.71 [–1.28, –0.14]). Dropout rates for any reason did not differ between two groups, but dropout rates due to side effects were significantly lower in Ginkgo biloba groups compared with placebo groups (OR = 1.72 [1.06, 2.80]).

**Conclusions:**

Taking a 240-mg daily dose of Ginkgo biloba extract is effective and safe in the treatment of dementia.

## Background

The standardized Ginkgo biloba extract EGb761 (Anatomical Therapeutic Chemical [ATC] code N06DX02) is classified as a therapeutic agent for dementia along with cholinesterase inhibitors and memantine in the ATC classification. Commission E of Germany [1], a committee that evaluates the efficacy and safety of herbal preparations, recognizes EGb761 as a medicine. The criteria for recognition as a medicine are improvement of symptoms, such as disturbance of memory, lack of concentration, depression, dizziness, tinnitus, etc., due to degenerative dementia, vascular dementia, or menopausal disorders. The standard composition of EGb761 preparations is 22.0% to 27.0% flavonoids and 5.0% to 7.0% terpenoids (ginkolides A, B, C; bilobalide; etc.) as active ingredients, and less than 5 ppm of ginkgolic acid, which is an allergen. Flavonoids inactivate deleterious toxic active oxygen, and terpenoids act as antagonists of platelet activating factor and exert neuroprotection in the brain [1,2]. By combining these pharmacological activities, Ginkgo biloba is thought to improve memory and learning ability, blood flow in the microcirculation, hypoxia tolerance in brain cells, and blood viscosity due to its antioxidant, antiinflammatory, and other activities.

Although Ginkgo biloba extract has been reported to be effective in the treatment of vertigo [3], tinnitus [4], headache [5], and anxiety disorders [6] in clinical trials, consistent, conclusive results were not reported because of small sample sizes in many. Although there are a few meta-analyses [7-10] and a Cochran review [11] in the literature, they mainly focused on the efficacy of Ginkgo biloba rather than safety, except for the reviews by Jiang et al. [10] and Birks and Evans [11]. They evaluated multiple measures of cognitive outcome by extracting the results of the ADAS-Cog, SKT, etc. to determine efficacy.

Ginkgo biloba extract is sold as a health food product in Japan and as an over-the-counter (OTC) preparation in Germany. It would be safer if it were also sold as an OTC preparation under the management of healthcare professionals such as pharmacists in Japan. For the safe use of Ginkgo biloba extract products from the viewpoint of both patients and healthcare professionals, the risk-and-benefit balance is important. The aim of this study was therefore to evaluate both the efficacy based on a single measure of cognitive outcome and safety based on various outcomes of Ginkgo biloba in the treatment of dementia using the meta-analysis approach.

## Methods

### Data sources

To identify relevant clinical studies, an electronic search was conducted using MEDLINE (1966–January 2014), Embase (1974–January 2014), the Cochrane Library (Issue 1 of 12, January 2014), and Japana Centra Revuo Medicina (Ichushi) (1981–September 2014). The terms and study design used in the searches were “Ginkgo,” or “*icho*” (in Japanese), “Alzheimer disease,” “cognitive defect,” “dementia,” and “multiinfarct dementia,” limited to “randomized controlled trial.” In Ichushi, original articles and randomized controlled trials were searched under the type of article and study design. We imposed no language limitation in the searches. Additionally, a manual search of reference listings from all of the articles retrieved from the electronic databases was performed.

### Inclusion criteria

The inclusion criteria of articles were that the studies: 1) were designed as double-blind, randomized, placebo-controlled trials; 2) had similar patients, study endpoints, dosage and administration, route of administration, duration of administration, etc.; 3) included patients with Alzheimer’s disease (AD), vascular dementia (VaD), mixed dementia with AD and VaD, and mild dementia. The standard mean difference (SMD) in the average value of the Syndrome Kurztest (SKT) [12] and Alzheimer’s Disease Assessment Scale cognitive subscale (ADAS-Cog) [13] scores was used as the efficacy endpoint. The dropout rate and incidence of adverse events were used as safety endpoints. The primary outcome was the SMD of the average value of cognitive measures consisting of the SKT and ADAS-Cog, and dropout rate due to adverse events, respectively. Secondary outcomes included subanalysis of disease classification, dose, and administration duration for efficacy endpoints and dropout rate by reason and dose and incidence of adverse events for safety endpoints.

Three investigators (MH, YO, MS) applied the inclusion criteria independently to the articles retrieved. When disagreements occurred, all three conferred to arrive at a consensus. The quality of the studies retrieved was assessed using the Jadad score [14]. Only studies that received a score of 3 points or more were included in the meta-analysis. The three investigators scored the studies independently. When there was discordance among the quality scores reported by the three investigators, the lowest score was adopted.

### Data analyses

In combing the data, efficacy in the treatment of dementia as evaluated by the SKT or ADAS-Cog in patients receiving Ginkgo biloba relative to those receiving placebo was calculated using the SMD [15]. Data were expressed as the SMD with 95% confidence intervals (CI). We adopted the SMD rather than odds ratio (OR) because the SMD represents the actual difference in the antidementia effect, i.e., in the SKT or ADAS-Cog score as a clinical outcome endpoint, thus giving a more realistic picture of the impact of improvement as a clinically observable outcome. A negative value of the pooled SMDs indicates greater improvement of dementia symptoms in patients who received Ginkgo biloba therapy relative to those who did not, whereas a positive value indicates the opposite. The statistical significance of differences between groups was evaluated using the 95% CI. Standard errors and CIs were converted to standard deviation for the analyses. Apart from the SMD, side effects were analyzed in terms of ORs with 95% CIs. For the meta-analysis, heterogeneity among studies was assessed using Q statistics, where a P value less than 0.05 was considered significant. When no significant heterogeneity was observed regarding the risk estimates among the studies, the fixed-effects model was applied for further analysis [15]. When significant heterogeneity was observed, the random effects model was applied [15]. Calculations of pooled SMDs with 95% CIs, as well as ORs with 95% CIs, were performed with Review Manager 5.2 (Cochrane Collaboration, Oxford, UK). The heterogeneity of effect size among studies was tested. To assess potential publication bias, funnel plots for each outcome were also examined.

## Results

**Figure 1:**
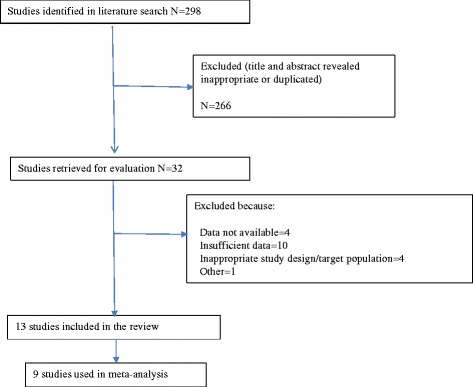
**Study selection for inclusion in the present meta-analysis.**

Among the 298 studies retrieved from the electronic databases and those retrieved from their references by manual search, we identified 13 clinical studies that satisfied the predetermined inclusion criteria for our study (Figure 1, Table 1) [16-28]. While no language limitation for the systematic search was set, all relevant articles meeting the inclusion criteria were published in English. One group of authors divided the data from the same study and published them in four articles, as shown in Table 1. Finally, 9 clinical studies were used in the meta-analysis. All used EGb761, the standard extract of Ginkgo biloba, as the study drug, and the duration of administration ranged from 12 to 52 weeks; in 5 studies it was administered for 24 weeks. The daily dose ranged from 120 to 240 mg: 120 mg in 2 studies; 160 mg in 2 studies; and 240 mg in 7 studies. Three studies included only AD, 5 included both AD and VaD, and 1 included dementia excluding AD and VaD. The clinical outcome endpoint was the SKT score in 7 studies and ADAS-Cog subscale score in 2 studies.

**Table 1:** **Summary of clinical studies meeting the initial inclusion criteria for this meta-analysis**

**Study**	**No. of patients and treatment groups**	**Duration (weeks)**	**Ginkgo biloba dose (mg)**	**Age (y)** **(mean** **[SD])**	**Gender female (%)**	**Inclusion criteria**	**Endpoint**	**Baseline cognitive scale** **(mean [SD])**	**Jadad score**
Herrschaft et al. [16]	Total: 402 EGb 761: 200 Placebo: 202	24	EGb 761: 240 once daily	Ginkgo: 65.1 (8.8) Placebo: 64.9 (9.4)	Ginkgo: 139 (69.5) Placebo: 140 (69.3)	Probable AD by NINCDS-ADRDA Possible AD with cerebrovascular disorder by NINCDS-AIREN Probable VaD by NINCDS-AIREN SKT: 9–23 (moderate dementia), HAMD <20 (except for serious depression)		Ginkgo: 15.1 (4.1) Placebo: 15.3 (4.2)	4
Ihl et al. [17]	Total: 404 EGb 761: 202 Placebo: 202	24	EGb 761: 240 once daily	Ginkgo: 65.0 (10.0) Placebo: 65.0 (9.0)	Ginkgo: 139 (69) Placebo: 133 (66)	Probable AD by NINCDS-ADRDA Possible AD with cerebrovascular disorder by NINCDS-AIREN Probable VaD by NINCDS-AIREN SKT: 9–23 (mild–moderate dementia), MMSE: 14–25, ADAS-Cog: 17–35		Ginkgo: 16.7 (3.9) Placebo: 17.2 (3.7)	4
Ihl et al. [18]	Total: 404 EGb 761: 202 (AD 163, VaD 39) Placebo: 202 (AD 170, VaD 32)	24	EGb 761: 240 once daily	AD: Ginkgo: 64.9 (9.5) Placebo: 64.2 (8.7) VaD: 65.8 (10.0) Placebo: 66.5 (10.7)	AD: Ginkgo: 109 (66.9) Placebo: 111 (65.3) VaD: Ginkgo: 30 (76.9) Placebo: 22 (68.8)	Probable AD by NINCDS-ADRDA Possible AD with cerebrovascular disorder by NINCDS-AIREN Probable VaD by NINCDS-AIREN SKT: 9–23 (mild–moderate dementia), MMSE: 14–25, ADAS-Cog: 17–35	SKT	AD: Ginkgo: 16.4 (3.8) Placebo: 17.0 (3.8) VaD: Ginkgo: 17.8 (3.9) Placebo: 18.3 (3.4)	4
Napryeyenko et al. [19]	Total: 395 EGb 761: 198 Placebo: 197	22	EGb 761: 240 twice daily	Ginkgo: 65.0 (8.0) Placebo: 63.0 (8.0)	Ginkgo: 143 (72) Placebo: 142 (72)	Probable AD by NINCDS-ADRDA Possible AD with cerebrovascular disorder by NINCDS-AIREN Probable VaD by NINCDS-AIREN SKT: 9–23 (mild–moderate dementia), MMSE: 14–25, ADAS-Cog: 17–35		Ginkgo: 15.6 (3.9) Placebo: 15.4 (3.7)	5
Napryeyenko et al. [20]	Total: 400 EGb 761: 200 (AD 106, VaD 94; Dropped, AD 2) Placebo: 200 (AD 112, VaD 88; Dropped, AD 2, VaD 1)	22	EGb 761: 240 twice daily	AD: Ginkgo: 66.0 (8.0) Placebo: 64.0 (8.0) VaD: Ginkgo: 63.0 (8.0) Placebo: 63.0 (9.0)	AD: Ginkgo: 70 (67) Placebo: 78 (71) VaD: Ginkgo: 73 (78) Placebo: 64 (74)	Probable AD by NINCDS-ADRDA Possible AD with cerebrovascular disorder by NINCDS-AIREN Probable VaD by NINCDS-AIREN SKT: 9–23 (mild–moderate dementia), MMSE: 14–25, ADAS-Cog: 17–35		AD: Ginkgo: 16.4 (3.8) Placebo: 15.8 (3.8) VaD: Ginkgo: 14.8 (3.8) Placebo: 15.0 (3.6)	5
Mazza et al. [21]	Total: 51 EGb: 25 Placebo: 26	24	EGb:160	Ginkgo: 66.2 (6.0) Placebo: 69.8 (3.0)	Ginkgo: 13 (52) Placebo: 16 (61)	AD by DSM-IV SKT: 8–23, MMSE: 13–25, IQ >80, GDS <11 (except for serious depression)		Ginkgo: 16.45 (3.05) Placebo: 15.90 (3.86)	4
Kanowski et al. [22]	Total: 205 EGb 761: 106 Placebo: 99 AD: 158 EGb 761: 79 Placebo: 79	24	EGb 761: 240 twice daily	Ginkgo: 72.0 (10.0) Placebo: 72.0 (10.0)	Ginkgo: 72 (68) Placebo: 70 (71)	AD or multiinfarct dementia by DSM-III-R SKT: 6–18, MMSE: 13–25		Ginkgo: 10.5 (3.2) Placebo: 11.2 (3.3)	4
van Dongen et al. [23]	Total: 123 EGb 761 (240 mg): 39 EGb 761 (160 mg): 40 Placebo: 44	24	EGb 761: 240 (high dose) 160 (low dose)	Ginkgo: 82.6 (5.1) Placebo: 82.5 (5.8)	Ginkgo: 68 (86) Placebo: 36 (82)	AD, VaD, mixed type, memory disorder with aging, early dementia patients (comparatively slight dementia, if the effect of Ginkgo biloba is marked) by DSM-III-R, ICD-10 SKT: 8–23, GDS <11 (except for serious depression), IQ >80, MMSE: 9–26		Ginkgo: 15.6 (4.1) Placebo: 14.1 (4.6)	4
Maurer et al. [24]	Total: 18 EGb 761: 9 Placebo: 9	12	EGb 761: 240	Ginkgo: 68.5 (6.0) Placebo: 60.6 (8.82)	Ginkgo: 5 (56) Placebo: 4 (44)	AD, probable AD by DSM-III-R, NINCDS-ADRDA		Ginkgo: 19.67 (6.31) Placebo: 18.11 (9.43)	4
Schneider et al. [25]	Total: 513 EGb 761 (240 mg): 170 EGb 761 (120 mg): 169 Placebo: 174	26	EGb 761: 240 120 twice daily	Ginkgo: 240 mg: 78.1 (7.0) 120 mg: 78.6 (7.0) Placebo: 77.5 (7.4)	Ginkgo: 240 mg: 96 (56) 120 mg: 84 (50) Placebo: 90 (52)	AD by DSM-IV Probable AD by NINCDS-ADRDA MMSE: 10–24 (to prevent excess evaluation, ≥20 not included)		Ginkgo (240 mg): 24.8 (12.7) Ginkgo (120 mg): 24.7 (11.9) Placebo: 25.01 (11.1)	5
Le Bars et al. [26]	Total: 309 EGb: 155 Placebo: 154	26, 52	EGb: 120	Ginkgo: 69.0 (10.0) Placebo: 69.0 (10.0)	Ginkgo: 79 (51) Placebo: 87 (56)	Uncomplicated AD or multiinfarct dementia by DSM-III-R, ICD-10 AD MMSE: 9–26, GDS: 3–6		Ginkgo: 20.0 (16.0) Placebo: 20.5 (14.7)	3
Le Bars et al. [27]	Total: 309 (AD: 236) EGb 761: 155 (AD: 120) Placebo: 154 (AD: 116) At 26-week endpoint: EGb 761: 136 Placebo: 134	26	EGb 761: 120	All: Ginkgo: 69.0 (10.0) Placebo: 69.0 (10.0) AD: 68.0 (10.0) Placebo: 68.0 (11.0)	All: Ginkgo: 79 (51) Placebo: 87 (56) AD: 65 (54) Placebo: 72 (62)	Uncomplicated AD or multiinfarct dementia by DSM-III-R, ICD-10 AD MMSE: 9–26, GDS: 3–6	ADAS- Cog	All: Ginkgo: 20.0 (16.0) Placebo: 20.5 (14.7) AD: Ginkgo: 19.7 (16.4) Placebo: 20.2 (15.2)	3
Le Bars et al. [28]	Total: 236 MMSE >23 EGb 761: 61 Placebo: 61 MMSE <24 EGb 761: 59 Placebo: 55	52	EGb 761: 120	MMSE >23: Ginkgo: 64.0 (9.0) Placebo: 64.0 (11.0) MMSE <24: Ginkgo: 73.0 (9.0) Placebo: 72.0 (9.0)	MMSE >23: Ginkgo: 34 (56) Placebo: 38 (62) MMSE <24: Ginkgo: 31 (53) Placebo: 34 (62)	Uncomplicated AD or multiinfarct dementia by DSM-III-R, ICD-10 AD MMSE: 9–26, GDS: 3–6		MMSE >23: Ginkgo: 9.0 (4.0) Placebo: 10.1 (4.9) MMSE <24: Ginkgo: 31.1 (17.0) Placebo: 31.9 (14)	3

NINCDS-ADRDA: Diagnosis criteria by the National Institute of Neurological and Communicative Disorders and Stroke (NINCDS) and the Alzheimer’s Disease and Related Disorders Association (ADRDA) working group. AIREN: Diagnostic criteria for vascular dementia by the Association Internationale pour la Recherche et 1’Enseignement en Neurosciences (AIREN). HAMD: Hamilton Rating Scale for Depression. MMSE: Mini-Mental State Examination. IQ: intelligence quotient. GDS: Geriatric Depression Scale.

The quality of each article was evaluated according to the Jadad scoring system (Table 1) [14]. All 13 clinical studies were assessed as high quality (>3 points), and 9 were included in the present meta-analysis, excluding the 4 reporting duplicated data.

**Figure 2:**
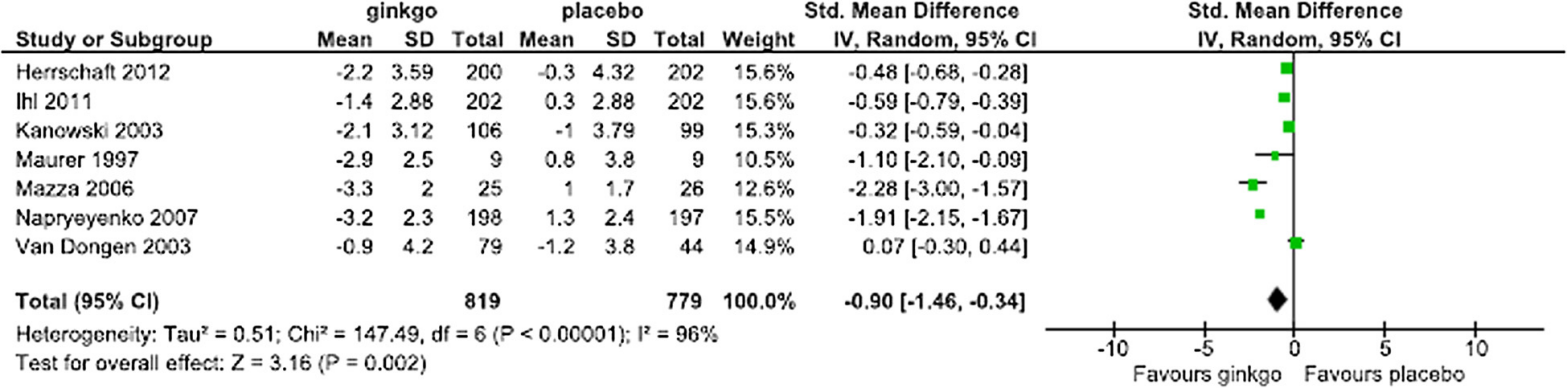
**Meta-analysis comparing Ginkgo biloba with placebo in the SKT in all patients.**

**Figure 3:**
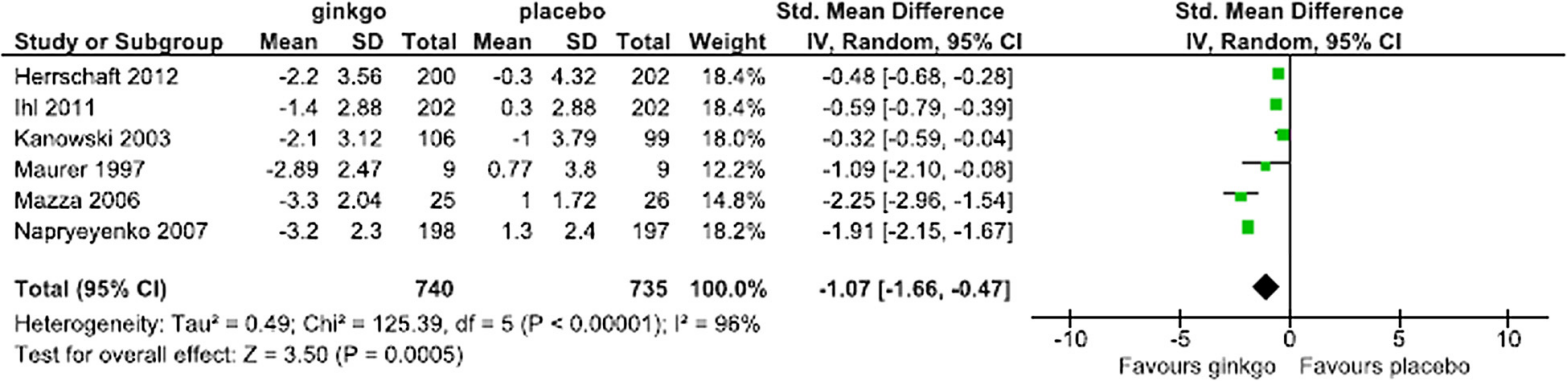
**Meta-analysis comparing Ginkgo biloba with placebo in the SKT in combined AD and VaD.**

**Figure 4:**
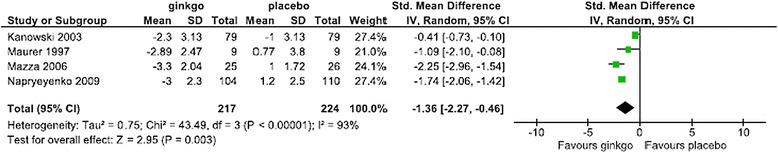
**Meta-analysis comparing Ginkgo biloba with placebo in the SKT in AD.**

**Figure 5:**
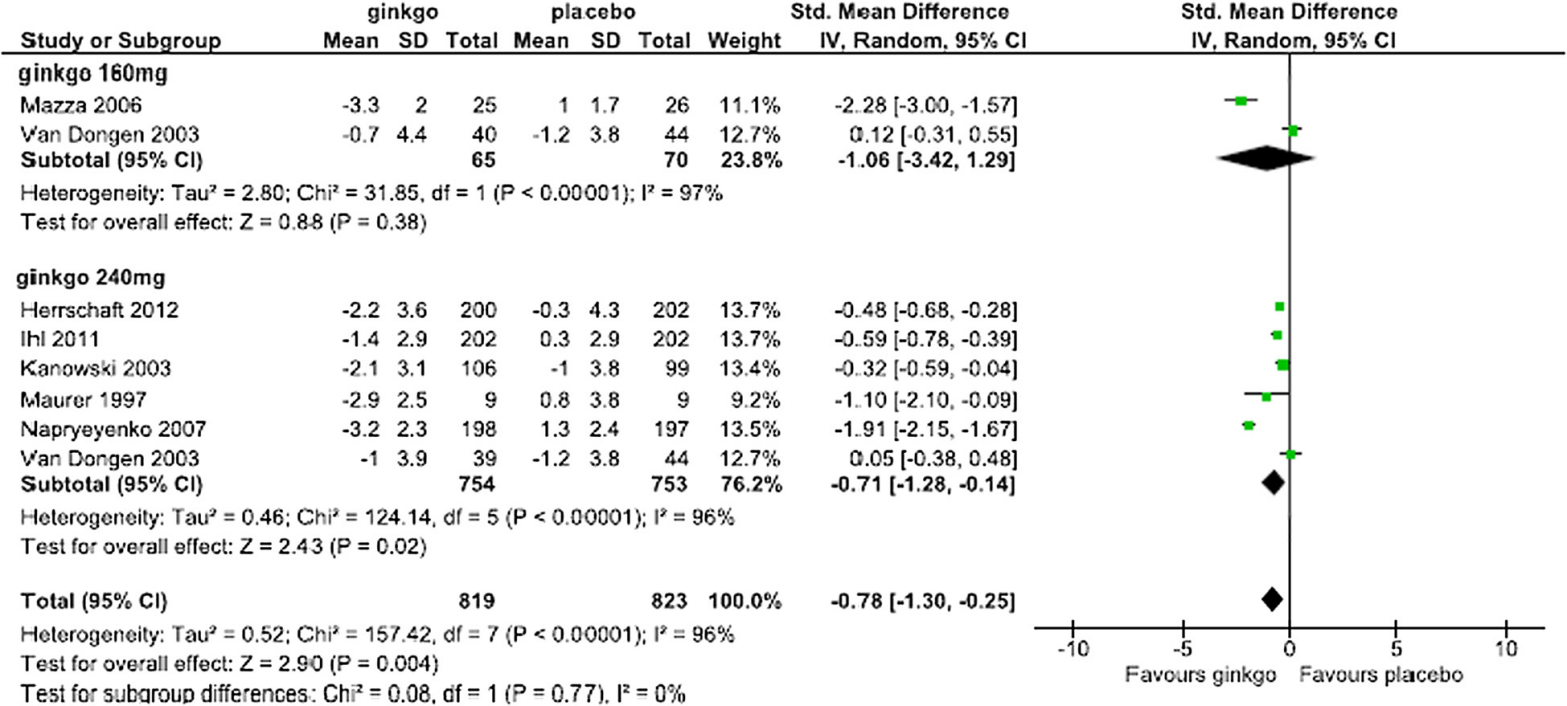
**Meta-analysis comparing Ginkgo biloba with placebo in the SKT at doses of 160 and 240 mg.**

**Figure 6:**
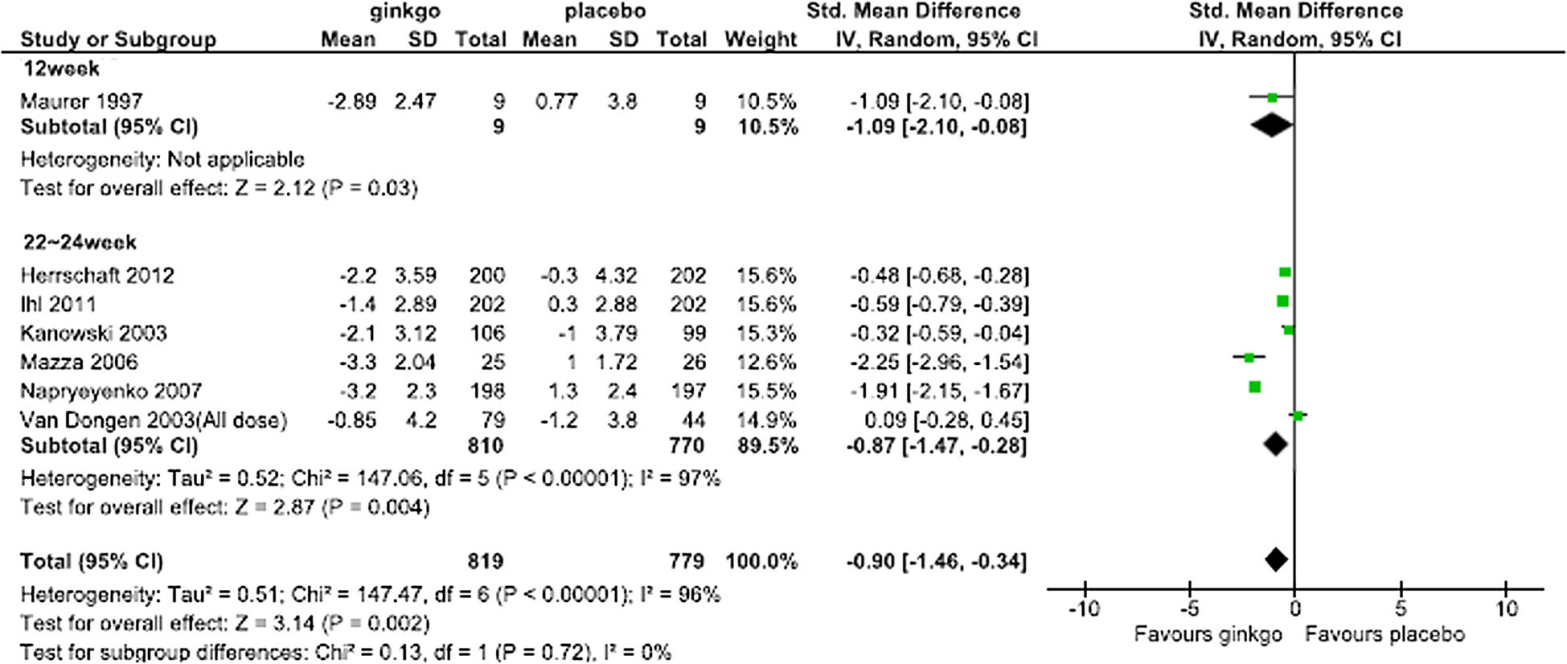
**Meta-analysis comparing Ginkgo biloba with placebo in the SKT at 12 weeks and 22–24 weeks of administration.**

For the SMD in the SKT, Forest plots of data from all patients, patients in the combined AD and VaD groups, patients in the AD-alone groups, subanalysis by dose, and subanalysis by the duration of administration are shown in Figures 2,3,4,5 and 6, respectively. The change in scores for the SKT in all patients ranged from –3.3 to –0.9 in the Ginkgo biloba groups and from –1.2 to 1.3 in the placebo groups. The SMD (95% CI) for the SKT in all patients were significantly greater for Ginkgo biloba than for placebo groups (–0.90 [–1.46, –0.34]) (Figure 2). The change in SKT scores in combined AD and VaD ranged from –3.3 to –1.4 in the Ginkgo biloba and from –1.0 to 1.3 in the placebo groups. The SMD for the SKT in combined AD and VaD was significantly greater for Ginkgo biloba than for placebo groups (–1.07 [–1.66, –0.47]) (Figure 3). Changes in scores in the SKT in AD ranged from –3.3 to –2.3 in the Ginkgo biloba groups and from –1.0 to 1.2 in the placebo groups. The SMD in the SKT in AD was significantly greater for Ginkgo biloba than for placebo (–1.36 [–2.27, –0.46]) (Figure 4). By dose, the change in SKT scores at 160 mg/day dose ranged from –3.3 to –0.7 in the Ginkgo biloba and from –1.2 to 1.0 in the placebo groups. The SMDs in the SKT at 160 mg/day were greater for Ginkgo biloba than for placebo (–1.06 [–3.42, 1.29]). At 240 mg/day, the SMDs in the SKT ranged from –3.2 to –1.0 in the Ginkgo biloba and from –1.2 to 1.3 in the placebo groups. The SMDs in the SKT at a dose of 240 mg/day were significantly greater for Ginkgo biloba than for placebo (–0.71 [–1.28, –0.14]) (Figure 5). By treatment duration, the changes in SKT scores at 12 weeks was –2.89 in the Ginkgo biloba and 3.8 in the placebo groups. The SMD in the SKT at 12 weeks was greater for Ginkgo biloba than for placebo (–1.09 [–2.10, –0.08]), and at 22–24 weeks ranged from –3.3 to –0.85 in the Ginkgo biloba and from –1.2 to 1.0 in the placebo groups. The SMDs in the SKT at 22–24 weeks were significantly greater for Ginkgo than for placebo (–0.87 [–1.46, –0.34]) (Figure 6).

**Figure 7:**
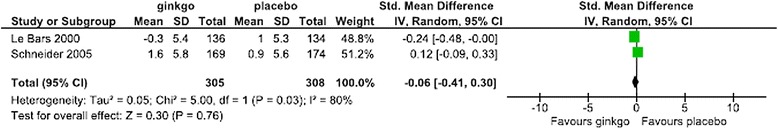
**Meta-analysis comparing Ginkgo biloba with placebo in the ADAS-Cog at 26 weeks at a dose of 120 mg.**

A Forest plot of the meta-analysis of the SMD in the ADAS-Cog at the dose of 120 mg evaluated at 26 weeks is shown in Figure 7. The change in scores for the ADAS-Cog ranged from –0.3 to 1.6 in the Ginkgo biloba and from 0.9 to 1.0 in the placebo groups. The SMD in the SKT at 26 weeks and 120 mg/day were greater for Ginkgo biloba than for placebo (–0.06 [– 0.41, 0.30]), but the difference did not reach statistical significance.

**Figure 8:**
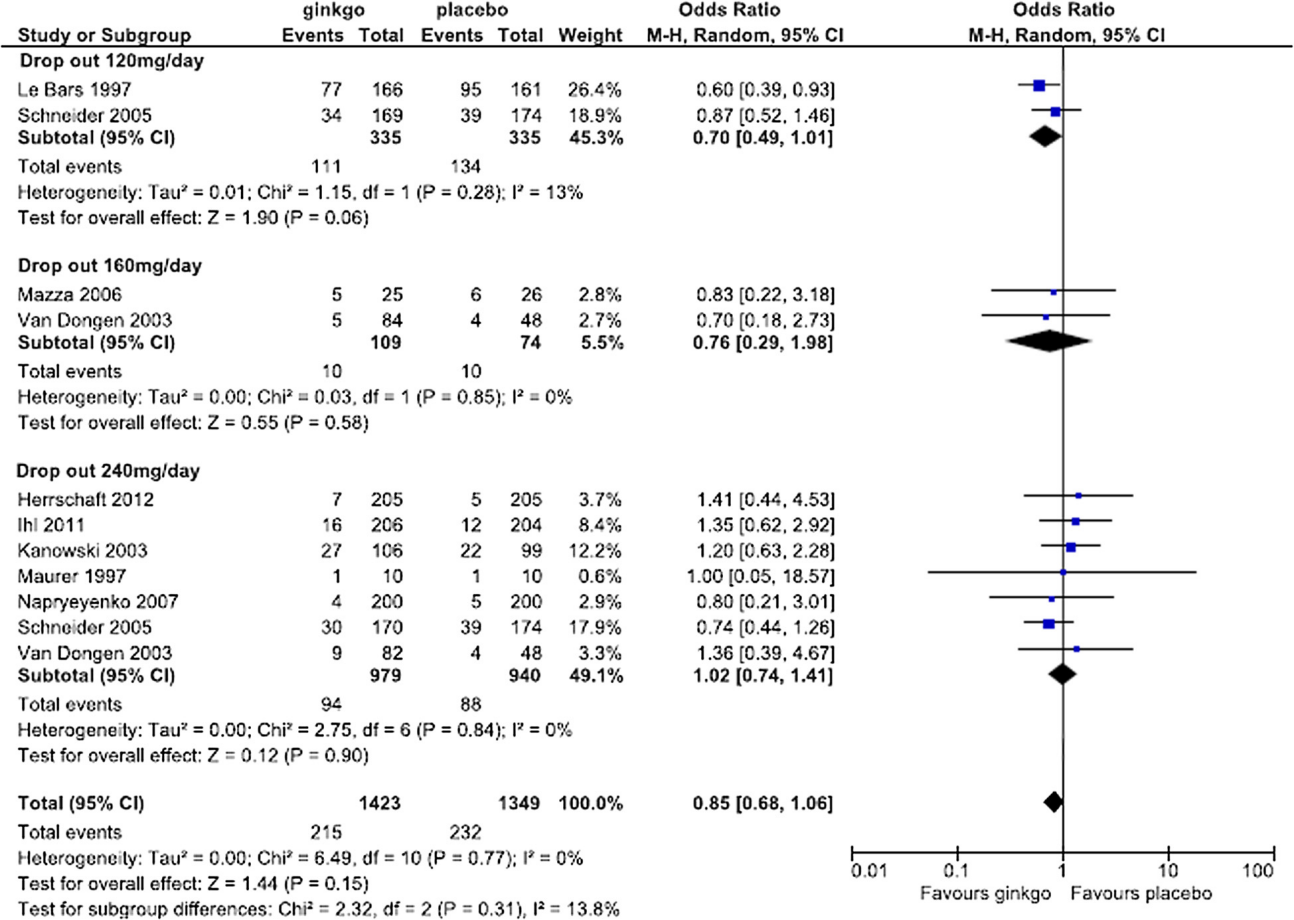
**Meta-analysis comparing Ginkgo biloba with placebo by dropout rate for any reason at doses of 120, 160, and 240 mg.**

**Figure 9:**
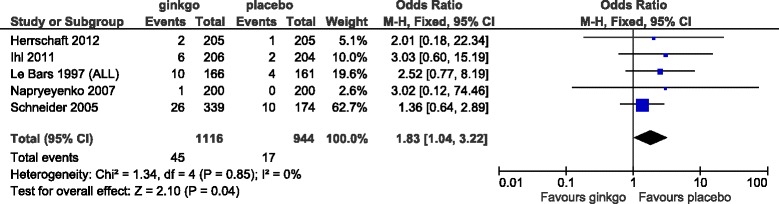
**Meta-analysis comparing Ginkgo biloba with placebo by dropout rate due to side effects.**

**Figure 10:**
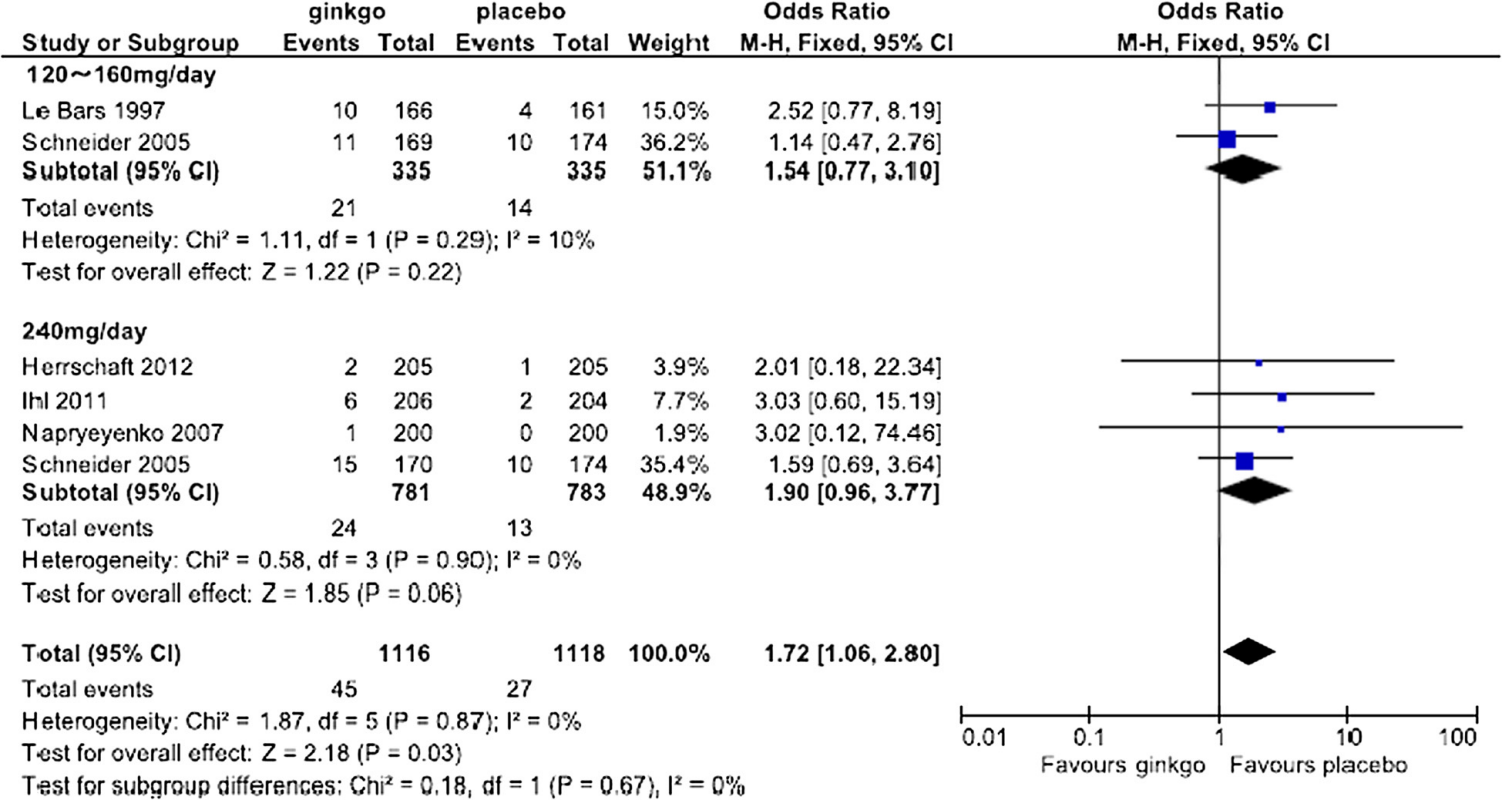
**Meta-analysis comparing Ginkgo biloba with placebo by dropout rate due to side effects at doses of 120–160 and 240 mg.**

For the safety analysis, the data of side effect with an incidence of at least more than 3% from initiation of treatment within 2 days after completion of treatment described in each report was extracted and analyzed. A Forest plot of the dropout rate for any reason and subanalysis by dose are shown in Figure 8. The change in the OR (95% CI) for the dropout rate was lower for Ginkgo biloba than for placebo (OR = 0.85 [0.68, 1.06]) but was not statistically significant. Subanalysis showed that the change in the OR for the dropout rate for any reason by dose was lower for Ginkgo biloba than for placebo (OR = 0.70 [0.49, 1.01] at 120 mg/day and OR = 0.76 [0.29, 1.98] at 160 mg/day), but the difference was not statistically significant. At the dose of 240 mg/day, Ginkgo biloba and placebo were nearly equivalent (OR = 1.02 [0.74, 1.41]). Forest plots of the dropout rate due to side effects and subanalysis by dose are shown in Figures 9 and 10, respectively. The change in the OR for the dropout rate due to side effects was significantly higher for Ginkgo biloba than for placebo (OR = 1.83 [1.04, 3.22]). Subanalysis of the change in the OR for the dropout rate due to side effects and dose was also higher for the Ginkgo biloba than for the placebo groups (OR = 1.54 [0.77, 3.10] at 120–160 mg/day and OR = 1.90 [0.96, 3.77] at 240 mg/day), but the difference was not statistically significant.

**Figure 11:**
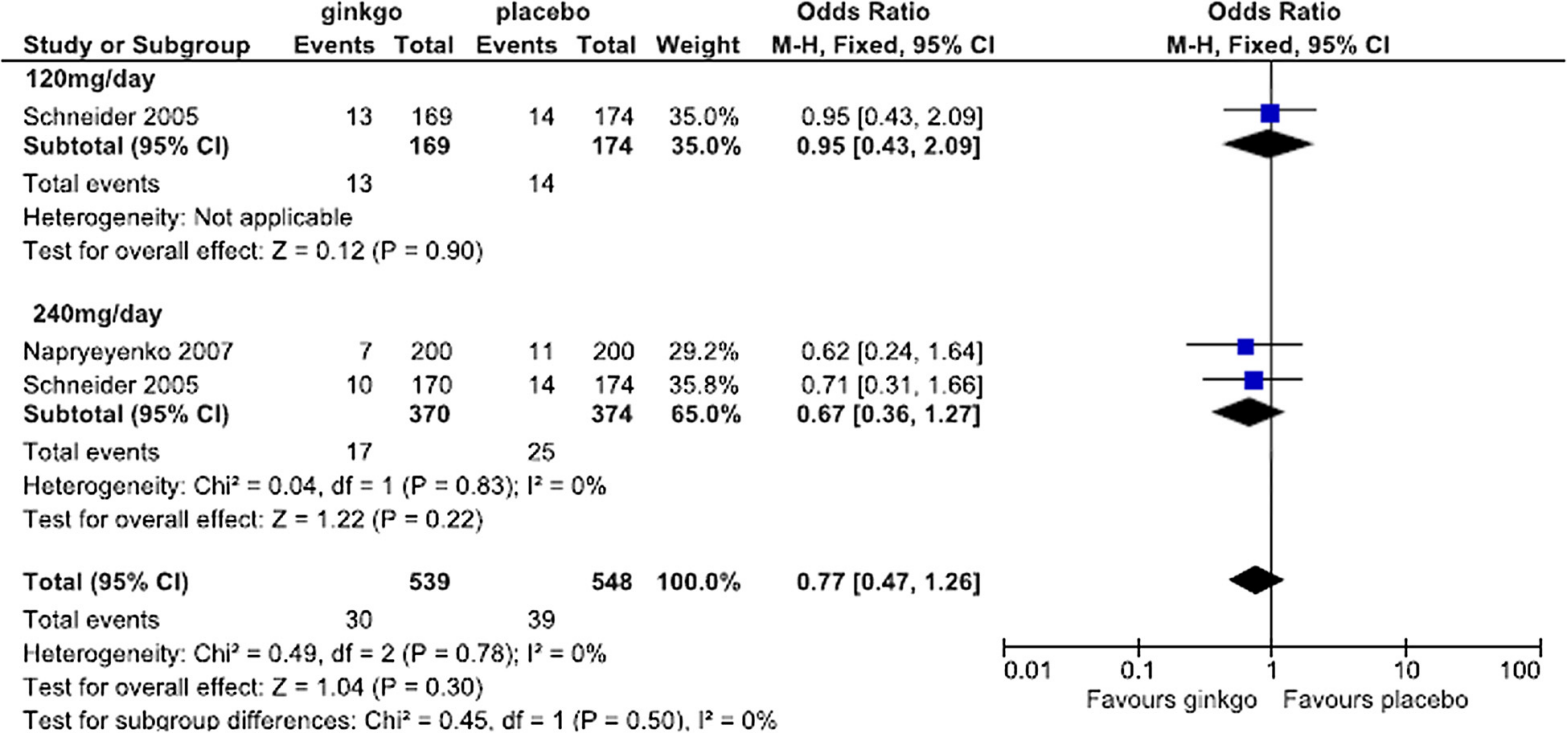
**Meta-analysis comparing Ginkgo biloba with placebo in the occurrence of severe side effects.**

**Figure 12:**
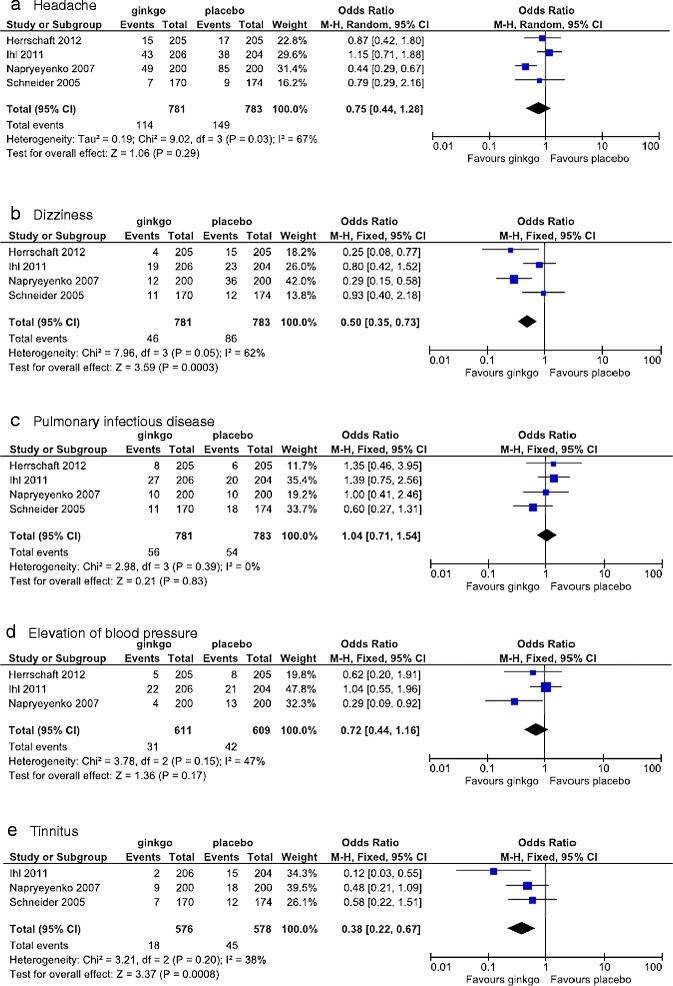
**Meta-analysis comparing Ginkgo biloba with placebo in the occurrence of specific side effects. a**: Headache**; b**: Dizziness; **c**: Pulmonary infectious disease; **d**: Elevation of blood pressure; **e**: Tinnitus.

The number and type of severe side effects are shown in Figure 11, and the incidence of side effects reported in more than 3% of patients is given in Figure 12. Headache, dizziness, and pulmonary infections occurred in 4 studies, and elevation of blood pressure and tinnitus in 3. Meta-analysis was performed for each of these side effects. The change in the OR for the incidence of severe side effects was lower for the Ginkgo biloba than for the placebo groups (OR = 0.77 [0.47, 1.26]), but the difference did not reach statistical significance. Subanalysis of the change in the OR by daily dose showed that Ginkgo biloba and placebo were nearly equivalent (OR = 0.95 [0.43, 2.09] at 120 mg/day). The change in the OR at the dose of 240 mg/day was lower for Ginkgo biloba than for placebo (OR = 0.67 [0.36, 1.27]), but not statistically significant. By specific side effects, the change in the OR for the incidence of headache was lower for Ginkgo biloba than for placebo (OR = 0.75 [0.44, 1.28]), but not significantly (Figure 12). The incidence of dizziness was significantly lower in the Ginkgo biloba than in the placebo groups (OR = 0.50 [0.35, 0.73]) (Figure 12). The incidence of infectious pulmonary disease was almost the same in the 2 groups (OR = 1.04 [0.71, 1.54]), with no statistically significant difference (Figure 12). Although elevation of blood pressure occurred less frequently in the Ginkgo biloba than in the placebo groups (OR = 0.72 [0.44, 1.16]), the difference was not significant (Figure 12). However, the incidence of tinnitus was significantly lower in the Ginkgo biloba than in the placebo groups (OR = 0.38 [0.22, 0.67]) (Figure 12).

Funnel plots for each outcome showed that there was no publication bias that could have affected the results of this meta-analysis.

## Discussion

Many clinical studies and several meta-analyses have shown the efficacy of Ginkgo biloba for the treatment of neuropsychiatric disorders, but almost all previous meta-analyses evaluated multiple measures of cognitive outcome by extracting the ADAS-Cog, SKT, etc. results as a marker of efficacy and did not evaluate the detailed safety outcomes. Therefore, a final conclusion has not been reached on its benefits as a medicine based on the balance between efficacy and safety. For the safe use of Ginkgo biloba extract products from the viewpoint of both patients and healthcare professionals, it is important to know the risk-and-benefit balance and the most effective method of administration in patients with dementia. By evaluating both the efficacy by a single measure of cognitive outcome and safety by detailed safety outcomes of Ginkgo biloba, this meta-analysis yielded results similar to previous ones and suggested that Ginkgo biloba has therapeutic potential for the treatment of dementia.

Thirteen clinical studies retrieved from the literature satisfied the predetermined inclusion criteria, and 9 were used in the present meta-analysis, excluding 4 reports based on the same data. Previous meta-analyses included 6–9 clinical studies of dementia. Disease classification in our meta-analysis included 3 reports on AD alone, 5 on combined AD and VaD, and 1 report on other dementia. The meta-analysis results for the SMD in the SKT between Ginkgo biloba and placebo in all patients, the AD and VaD combined group, and AD-alone group showed a statistically significant difference favoring Ginkgo biloba over placebo. Because a higher SKT score indicates a more severe level of dementia [29], these results suggest that Ginkgo biloba can improve the symptoms of AD and/or VaD. They were generally consistent with the results of the Cochrane review reported in 2009, in which the SMD was –1.30 (95% CI: –3.10, 0.50] [11].

When conducting subgroup analysis by dose, the SMD (95% CI) in the SKT at 160 mg and 240 mg were –1.06 (–3.42, 1.29) and –0.71 (–1.28, –0.14), respectively, with a statistically significant difference only between the latter and placebo. Jiang et al. [10] reported a similar result when comparing changes in cognitive scores in patients who received Ginkgo biloba >200 mg/day and <200 mg/day. The effects of Ginkgo biloba on the central nervous system as measured in the brain wave test suggested dose responsiveness [30], and the present results may partially support that although we found no clear dose-response difference between the doses of 160 mg and 240 mg daily.

There was no statistically significant difference in the ADAS-Cog results (–0.06 [–0.41, 0.30]) in our meta-analysis, although a tendency toward improvement was noted in the Ginkgo biloba group. Two studies included in this meta-analysis had similar patient numbers but conflicting ADAS-Cog results. A possible reason for this may have been differing diagnostic criteria and the dosage used. The dose in both of those studies was 120 mg daily, while our SKT results indicated the efficacy of Ginkgo biloba 240 mg daily. It appears likely that a full response was not achieved with a daily dose of 120 mg.

The dropout rate for any reason in all combined studies had an OR of 0.85 (95% CI: 0.67, 1.08) and the dropout rate at doses of 120 mg/day, 160 mg/day, and 240 mg/day, which showed efficacy in the SKT results, were 0.70 (0.49, 1.01), 0.76 (0.29, 1.98), and 1.02 (0.74, 1.41), respectively. None of the studies at doses of 120 mg/day, 160 mg/day, and 240 mg/day in this meta-analysis showed a statistically significant difference in dropout rates for any reason compared with placebo. The OR of the dropout rate due to side effects in all combined studies was 1.83 (1.04, 3.22) and there was a statistically significant difference between Ginkgo biloba and placebo. Although there was no significant difference between Ginkgo biloba and placebo in subanalysis of the doses of 120–160 mg/day and 240 mg/day, these data showed that the incidence of side effects in Ginkgo biloba groups tended to be higher than that in placebo groups. A tendency for a dose-response relationship in the dropout rate due to side effects was also suggested.

There was no significant difference between the Ginkgo biloba and placebo groups in the occurrence of severe side effects, in the incidence of side effects reported in 3 or more studies, or in side effects experienced by 3–5% of patients from the beginning of treatment until 2 days after the end of the studies analyzed. In the meta-analysis for each side effect including headache, pulmonary infection, and elevation of blood pressure, no significant differences were found between Ginkgo biloba and placebo. On the other hand, the cumulative OR (95% CI) for dizziness and tinnitus was 0.50 (0.35, 0.73) and 0.38 (0.22, 0.67), respectively, indicating a significantly lower incidence in the Ginkgo biloba compared with the placebo groups. Commission E of Germany also found that the treatment of dementia with Ginkgo biloba was not associated with the occurrence of dizziness and tinnitus. This study confirmed the preventive effect of Ginkgo biloba against dizziness and tinnitus. It is also known that many elderly people experience dizziness and tinnitus, and thus Ginkgo biloba preparations would be advantageous in preventing these side effects in those with dementia.

There were some limitations in this meta-analysis. Similar to the evaluation of other antidementia agents using the SMD at baseline and SKD and ADAS-Cog scores after treatment as clinical outcomes, it should be noted that the extent of treatment effect differs depending on baseline values. Although there was little difference in the baseline values in the studies included this meta-analysis, the study protocols, severity of disease, areas where the studies were performed, etc. differed. Therefore, we used the random-effect model in meta-analysis after determining the heterogeneity of the studies.

In summary, Ginkgo biloba appears more effective than placebo in the treatment of dementia at doses of more than 240 mg daily administered for 22 weeks when using the SKT as a cognitive outcome measure, and safety did not differ markedly between them.

## Conclusions

Taking a 240-mg daily dose of Ginkgo biloba extract is effective in the treatment of dementia. Ginkgo biloba extract is therefore useful in improving the symptoms of dementia, as found in previous reports of meta-analyses. The evidence for efficacy and safety found in the present meta-analysis of the effects of Ginkgo biloba extract contributes to knowledge of the treatment of dementia.
